# Uncommon manifestation of Peutz-Jeghers syndrome: a case of jejuno-jejunal intussusception and volvulus leading to small bowel obstruction

**DOI:** 10.1093/jscr/rjae335

**Published:** 2024-05-28

**Authors:** Hesham Barhmji, Abduraboh Alsalehi, Ahmad Kammasha, Ahmad Alkheder

**Affiliations:** General Medicine Department, Faculty of Medicine, Damascus University, Damascus 963, Syria; General Surgery Department, Maarib General Hospital Authority, Maarib 967, Yemen; General Surgery Department, Maarib General Hospital Authority, Maarib 967, Yemen; General Surgery Department, Pavol Jozef Safarik University, Kosice, Slovakia; Department of Otolaryngology – Head and Neck Surgery, Al-Mouwasat University Hospital, Faculty of Medicine, Damascus University, Damascus 963, Syria; Department of Otolaryngology – Head and Neck Surgery, Al-Mouwasat University Hospital, Faculty of Medicine, Damascus University, Damascus 963, Syria; General Medicine Department, Faculty of Medicine, Syrian Private University, Damascus 967, Syria

**Keywords:** Peutz-Jeghers syndrome, volvulus, intussusception, PJS, emergency, bowel obstruction

## Abstract

Peutz-Jeghers syndrome (PJS) is a rare genetic disorder causing gastrointestinal polyps and skin pigmentation. Our case report highlights a unique instance of jejuno-jejunal intussusception associated with PJS in a 28-year-old female patient who presented to the emergency department with colicky abdominal pain, tachycardia, and gastrointestinal symptoms. Physical examination revealed mucocutaneous hyperpigmentation. Imaging studies showed a U-shaped distension in the jejunum with thickening and pneumatosis. Laparotomy revealed a jejuno-jejunal volvulus with intussusception. Surgical resection successfully addressed gangrenous jejunal tissue and ileal polyps. Histopathology confirmed PJS polyps. Postoperatively, the patient recovered well and was discharged. Family history revealed similar skin lesions in her uncle. Our case highlights the need for prompt surgical intervention to address complications associated with PJS and elucidates a unique presentation of PJS involving jejuno-jejunal intussusception and volvulus leading to complete small bowel obstruction. We aim to deepen understanding and prompt discussions on optimal therapeutic strategies.

## Introduction

Peutz-Jeghers syndrome (PJS), a relatively rare autosomal dominant disorder with variable penetrance, manifests as the development of polyps within the gastrointestinal tract, particularly the colon and rectum, with only a scant number of documented cases in literature [1]. The incidence of PJS is estimated to range from 1 in 8300 to 200 000 births [2]. Initially described by Peutz in 1921, and later elucidated by Jeghers, McKusick, and Katz in 1949 [3–5], PJS is characterized by the triad of gastrointestinal polyps, mucocutaneous pigmentation, and familial predisposition [6]. Gastrointestinal involvement primarily manifests as hamartomatous polyps, predominantly found in the small bowel, colon, and rectum in over 90% of cases, with less frequent occurrences in the stomach or urinary tract [7]. These hamartomas typically measure between 5 and 50 mm in diameter, with a median size of 35 mm, and are associated with complications such as intussusception, bleeding, anemia, and obstruction [8]. Herein, we present an unusual case of jejuno-jejunal intussusception attributed to PJS in a female patient.

This work is also reported in line with Surgical Case Report criteria, which helped to improve the transparency and quality of this case report [9].

## Case presentation

**Figure 1:**
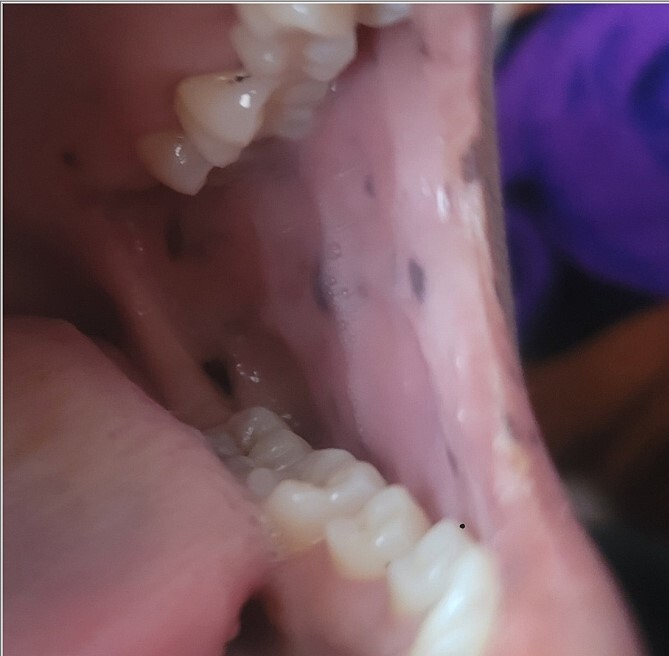
Mucocutaneous pigmentation on the buccal mucosa and lips.

**Figure 2:**
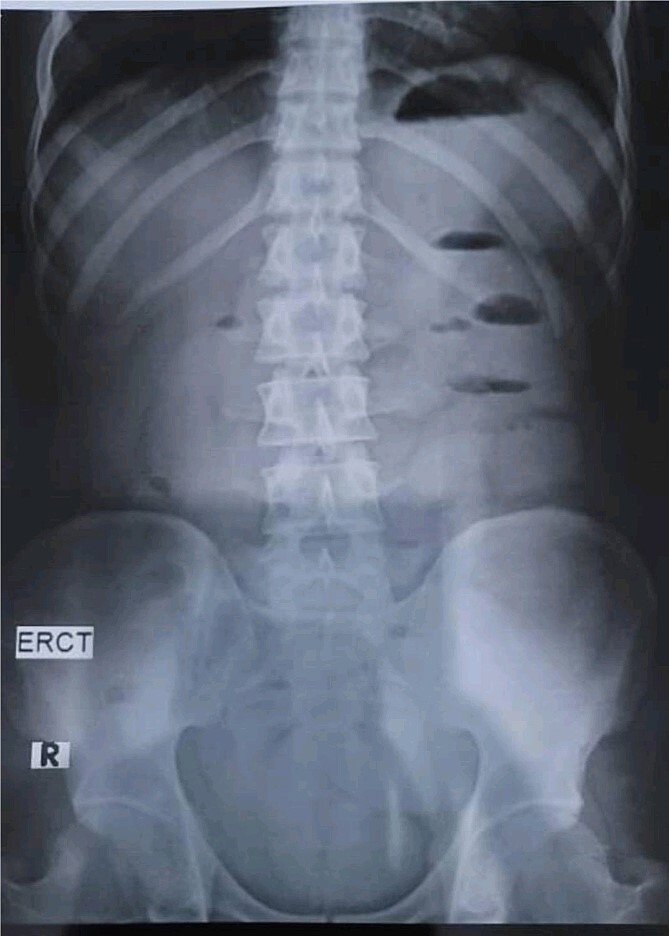
Abdominal X-rays in erect position reveals five air fluid levels.

A 28-year-old female patient arrived at the emergency department complaining of intermittent colicky abdominal pain and presenting with tachycardia. She had not passed stool or flatus for 3 days, accompanied by vomiting. Upon physical examination, mucocutaneous hyperpigmentation was observed in the oral cavity (Fig. 1), along with a distended abdomen and increased bowel movement, which were hypertympanic on percussion. Initial investigations, including abdominal and pelvic ultrasonography, abdominal X-rays in erect and supine positions (Fig. 2), chest X-ray and computed tomography scan, revealed a 3-cm diameter, a 5 air level fluid, a U-shaped distension with 3.2-cm diameter mural thickening, mesenteric fat infiltration, and pneumatosis. There were no signs of peritonitis, and laboratory values fell within normal limits.

**Figure 3:**
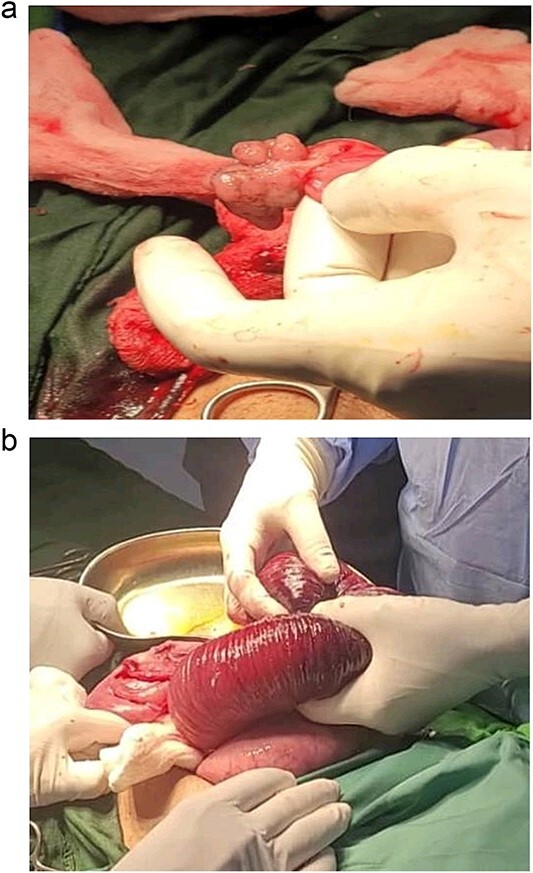
View during the surgery: intussusception jejuno-jejunal and hamartomatous polyps of the ileum.

**Figure 4:**
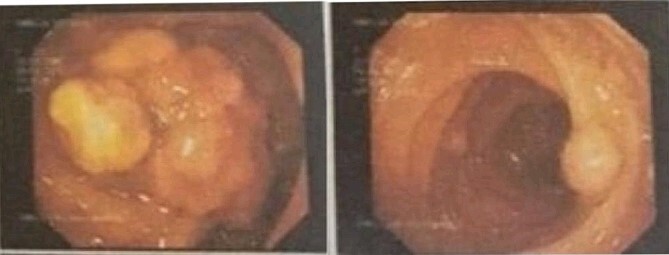
Colonoscopy with multiple polyps.

**Figure 5:**
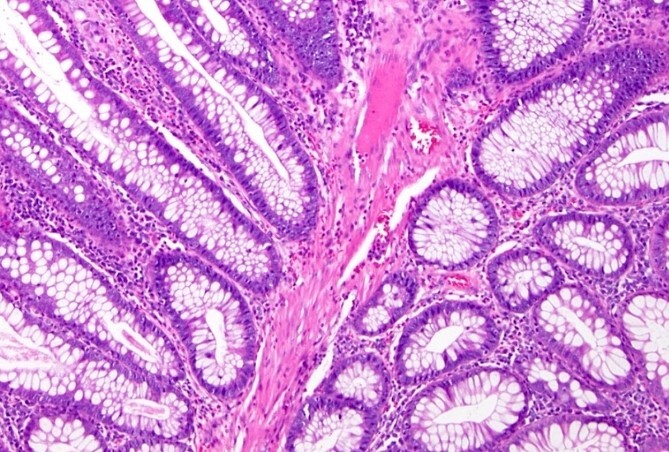
Histopathological study: showing an edematous appearance of the intestinal mucosa with inflammatory lymphocytes and histiocytes.

The decision was made to proceed with laparotomy. Intraoperatively, upon midline laparotomy, a jejuno-jejunal U-shaped volvulus with intussusception was initially identified (Fig. 3). Following multiple maneuvers to manually reduce the intussusception, ~80 cm of gangrenous jejunal intussusceptum was resected, followed by end-to-end anastomosis. Examination of other segments of the small and large bowel revealed two ileal polyps, with dimensions of ~3 × 5 cm^2^ and 2 × 2 cm^2^, respectively (Fig. 3). Each polyp was resected, and the surgical sites were repaired primarily. Following closure of the abdomen, the patient was transferred to the intensive care unit for postoperative monitoring. The excised mass was sent for histopathological analysis, which confirmed the presence of typical hamartomatous polyps. Postoperatively, the patient demonstrated satisfactory progress, tolerated oral feeding and was discharged on the seventh day after surgery. Two months later, diagnostic endoscopy revealed multiple polyps in the large bowel measuring 5–15 mm (Fig. 4), without evidence of intussusception. These polyps were removed via endoscopic polypectomy. Histopathological examination of the removed polyps confirmed the presence of characteristic PJS polyps, displaying a branching framework of connective tissue and smooth muscle lined by normal intestinal epithelium (Fig. 5). Subsequently, a detailed family history revealed similar skin lesions in the patient’s uncle.

## Discussion

PJS is an inherited disorder characterized by multiple distinctive polyps in the gastrointestinal tract, often accompanied by mucocutaneous pigmentation, particularly noticeable around the lips. It is inherited in an autosomal dominant manner, attributed to mutations in the STK11 (LKB1) gene [8].

The prevalence of PJS varies across studies, ranging from 1 in 8300 to 1 in 280 000 individuals, with probable prevalence of ~1 in 100 000 people. Diagnosis typically involves the presence of hamartomatous polyps alongside at least two of the following: labial melanin deposits, a family history of the syndrome and small bowel polyposis. PJS affects both genders equally and is observed across all ethnic groups [2, 6].

Clinical symptoms of PJS are characterized by periods of asymptomatic intervals punctuated by complications such as abdominal pain, recurrent intussusception leading to bowel obstruction, rectal polyp prolapse, and often occult bleeding. Small bowel obstruction is a common initial presentation, often necessitating repeat surgeries due to polyp-related complications occurring at relatively short intervals [7, 10, 11].

In addition to gastrointestinal polyps, PJS patients face an elevated risk of both gastrointestinal and non-gastrointestinal malignancies. Studies have shown a cumulative cancer risk of up to 93% between ages 15 and 64, with a relative risk of developing neoplasia up to 15 times higher than the general population. Colonic tumors are the most prevalent, followed by breast, pancreatic, gastric, ovarian, small intestinal, and uterine cancers [10, 12, 13].

Traditionally considered benign, the management of PJS has evolved towards a more proactive approach, advocating for the removal of all polyps upon histological confirmation to prevent complications like intussusception and subsequent bowel resection leading to short-bowel syndrome. Polypectomy is recommended for large polyps in the stomach or colon detected during endoscopic surveillance, while surgery is advised for symptomatic or rapidly growing small intestinal polyps or asymptomatic ones exceeding 1–1.5 cm. Techniques like intraoperative endoscopy with polypectomy or enterotomy are employed to facilitate complete polyp removal and reduce the need for recurrent surgeries [14].

In our presented case, the patient was admitted with intussusception, requiring exploratory laparotomy that revealed non-viable small bowel segments, necessitating resection and subsequent end-to-end anastomosis.

## Conclusion

In this case report, we shed light on a distinctive manifestation of PJS marked by jejuno-jejunal intussusception, volvulus, and subsequent complete small bowel obstruction. By delving into the diagnostic intricacies and surgical interventions employed, our objective is to enhance comprehension of this uncommon clinical phenomenon. We aspire for this case report to enrich the current body of literature and stimulate discourse regarding optimal therapeutic modalities for analogous presentations.
